# Assessing the cost‐effectiveness of interventions within a humanitarian organisation

**DOI:** 10.1111/disa.12344

**Published:** 2019-04-22

**Authors:** Chloe Puett

**Affiliations:** ^1^ Senior Research Advisor Action Against Hunger United States

**Keywords:** cost‐effectiveness analysis, humanitarian interventions, value for money

## Abstract

Cost‐effectiveness analysis is increasingly relevant in humanitarian action. The cost of response has increased exponentially in the past decade, alongside concurrent donor budget restrictions. However, there remains limited comprehension and application of these methods in this field. This paper documents methods developed for use within Action Against Hunger, an international humanitarian organisation, in response to a lack of understanding of this topic within the humanitarian community and limited evidence of the cost‐effectiveness of humanitarian action. These methods encompass costs to both implementing institutions and participating communities. Activity‐based cost analyses are conducted to assess resources per programme activity. Cost‐effectiveness is evaluated using successful programme outcomes, and uncertainty is appraised via sensitivity analysis. This paper aims to advance knowledge, stimulate discussion, and promote the adoption of cost‐effectiveness methods for building the evidence base for humanitarian action, including consideration of community costs, to enable analytical outputs that are useful for managers and policymakers alike.

## Introduction

Measures of intervention resource use are becoming increasingly important in humanitarian action. The cost of humanitarian response has risen exponentially over the past decade (Scott, 2015). In light of budget restrictions, donors are increasingly held accountable for their own investments by citizens, in the case of donor governments, along with other constituents, and humanitarian actors are asked to document the ‘value for money’ of humanitarian response (DFID, 2011). Yet, there is also limited evidence and understanding of the costs and cost‐effectiveness of humanitarian action, creating a demand for research to fill this gap.

There has been scant application to date of cost‐effectiveness analysis (CEA) in humanitarian action—examples include Griekspoor, Sondorp, and Vos (1999), Reithinger and Coleman (2007), and Gosselin, Maldonado, and Elder (2010). One possible reason for this is ethical concerns, such as traditional resistance to assessing the cost‐effectiveness of lifesaving aid. Another potential reason is a lack of clarity among the broader humanitarian community about how to apply economic methods, given the challenges of doing so in humanitarian settings (Hallam, 1996; Goyet et al., 2006).

### Methodological choices in CEA

The lack of application of CEA methods is not due to a lack of guidance. Several guidelines exist that outline recommended methods for economic analysis, including CEA (Russell et al., 1996; Tan‐Torres Edejer et al., 2003; Muennig, 2008). A CEA begins with several analytical choices, including which costs the analysis should encompass. Analyses can use either predicted or actual cost data. Predictive data are used when a cost estimate is needed for a new programme, and no actual cost data are available (Waters, 2000). Many CEAs utilise data from organisational accounting systems to document actual, rather than planned, programme resource use. There are many non‐budgeted costs, though, that contribute to the functioning of a programme. The World Health Organization (WHO) recommends that CEAs also include these ‘economic’ costs, rather than only financial costs (Johns, Baltussen, and Hutubessy, 2003). This approach is intended to capture all resources used by a programme, whether or not the implementing agency paid for them. This also means that an analysis must go beyond routinely documented accounting data, and develop a broader perspective on the costs of a programme. Capturing these costs may require additional data sources, such as interviews and surveys, depending on the desired level of detail and precision.

The choice of which costs to include depends largely on the analytical perspective. An assessment that adopts the ‘institutional’ perspective includes all costs incurred by the institutions delivering the programme. An analysis that adopts the societal perspective, as is recommended by the WHO‐CHOICE (CHOosing Interventions that are Cost‐Effective) project and the United States Panel on Cost‐Effectiveness in Health and Medicine, considers all of the costs of an intervention, regardless of who has incurred them (Russell et al., 1996; Tan‐Torres Edejer et al., 2003). Societal CEAs include direct costs such as travel expenses and indirect costs such as the value of time spent by household members participating in or accessing care from an intervention (Russell, Fryback, and Sonnenberg, 1999; Musgrove and Fox‐Rushby, 2006). Existing research in developing countries has demonstrated that these ‘hidden costs’, particularly distance from services and cost of travel, are important determinants of service utilisation, a key factor in effectiveness (Sauerborn, Nougtara, and Diesfeld, 1989; Floyd, Wilkinson, and Gilks, 1997; Nahar and Costello, 1998; Islam et al., 2002; Mirzoev et al., 2008; Ayieko et al., 2009; Saksena et al., 2010; Puett et al., 2013a).

Where actual programme expenditure data are available, it is possible to conduct micro costing analyses using an ‘ingredients’ approach, as is endorsed by WHO and the World Bank (Johns, Baltussen, and Hutubessy, 2003; Tan‐Torres Edejer et al., 2003; Horton et al., 2010). This method costs and quantifies each programme input, including personnel and supervision time, providing a detailed and transparent account of programme resource use (Johns, Baltussen, and Hutubessy, 2003). This allows analysts and policymakers to judge the appropriateness of cost estimation and to gauge whether costs from one analysis can be modified for use in another setting (Tan‐Torres Edejer et al., 2003). The limitations of this method are the potential for underestimating more ‘distal’ costs, such as higher‐level support costs, or omitting incurred costs that were unanticipated during programme design (Fiedler, 2003; Caldes, Coady, and Maluccio, 2006).

Costs should be categorised to facilitate analysis. Accounting cost centres traditionally are used to organise costs by input category, such as personnel, medical supplies, and transport. Costs can also be organised by the level at which they were incurred, including capital, district, or sub‐district (Tan‐Torres Edejer et al., 2003). Activity‐based costing uses programme activities as an intermediate step to allocate the total costs of a programme to its products and services. This method is particularly useful for allocating costs that are shared among various activities, programmes, or organisations (Waters, Abdallah, and Santillán, 2001; Fiedler, 2003; Waters et al., 2006; Fiedler, Villalobos, and De Mattos, 2008; Puett et al., 2013a).

### Expressing outcomes in CEA

The results of CEAs are expressed as a cost‐effectiveness ratio (CER), with total programme resources divided by ‘effectiveness’ with respect to outcomes achieved (Musgrove and Fox‐Rushby, 2006). Incremental analyses are used to compare two programmes with the same outcome, such as child recovered from acute malnutrition or death prevented, in terms of the difference in the costs and outcomes of the two interventions (Musgrove and Fox‐Rushby, 2006). The incremental cost‐effectiveness ratio (ICER), calculated as the difference in costs divided by the difference in outcomes of two interventions, represents the amount of resources needed to gain an additional outcome, or ‘unit of effectiveness’ (Muennig, 2008), by one programme compared to its next‐best alternative. The cost‐effectiveness of an intervention can also be compared to a ‘do nothing’ alternative, providing policymakers with information on the absolute costs and effects of a programme relative to no intervention, rather than incremental to another existing one. The calculation of CERs in this manner is commonly practised in CEAs for public health and nutrition programmes (Ashworth and Khanum, 1997; Islam et al., 2002; Bachmann, 2009).

Effectiveness can be expressed in terms of programme outcomes, such as case of acute malnutrition prevented or cured. Measures also can be used that enable comparisons of health effects between programmes addressing different health outcomes; these are based on the concept of ‘utility’, or the benefit to an individual of being in a particular health state. The two most common types of utility measures applied in ‘cost–utility’ analysis are quality‐adjusted life years (QALYs) and disability‐adjusted life years (DALYs), which are based on subjective weighting of health states (Gold, Stevenson, and Fryback, 2002). QALYs are a measure of health gains achieved according to patient self‐assessment, whereas DALYs are a measure of burden of disease according to clinical experts (Murray, 1994; Murray et al., 2001; Drummond et al., 2005; Muennig, 2008).

The major advantage of using outcome measures such as DALYs for cost–utility analysis is the comparability of measurement across disease states, allowing for analysis of comparative effectiveness among interventions addressing different health outcomes (Murray, 1994; Musgrove and Fox‐Rushby, 2006). Important limitations are that they can only be used for health outcomes, and they are based on several key assumptions about the severity and duration of a condition, age at onset, and remaining life expectancy at that age (Murray, 1994; Fox‐Rushby and Hanson, 2001; Musgrove and Fox‐Rushby, 2006). The impact of such assumptions on analytical outcomes can be attenuated to some extent by clearly stating assumptions and using sensitivity analysis to assess how such assumptions affect outcomes.

### Methods applicable to humanitarian interventions

Standard CEA methods can be applied across many kinds of interventions. However, in evaluating the cost‐effectiveness of humanitarian interventions, careful consideration of the principles underlying such interventions may enable the development of methods that either are more acceptable for humanitarian action or help to provide evidence that is useful for humanitarian programming. One humanitarian principle that may be viewed as being at odds with economic assessment is the ‘humanity’ principle, or the imperative to address human suffering wherever it is found (Leader, 2000). The humanitarian focus on protecting imperilled lives does not necessarily prioritise efficiency over timeliness and effectiveness of intervention, particularly where these may be at odds with one another.

Given the expanding interest in CEA, and the remaining gaps in evidence on the cost‐effectiveness of humanitarian action, the objective of this paper is to document the CEA methods and analytical process developed for use within a single global humanitarian organisation, Action Against Hunger | ACF International (ACF). This is intended to serve as both a resource for the international community, as well as to promote understanding, stimulate discussion, and advocate for broader adoption of CEA in this field.

## Methods

### Context

ACF is an international non‐governmental organisation working to prevent, detect, and treat malnutrition both in humanitarian emergencies and longer‐term programmes integrated into regional and national health systems. Interested in understanding CEA methods in light of increasing donor demands for economic analysis of their field programmes, ACF‐France started a research project on cost‐effectiveness in 2012. As part of this endeavour, ACF developed CEA methods, based on methods developed in prior research (Puett et al., 2013a), which take a societal perspective and employ mixed methods and activity‐based costing. These methods initially were applied in two analyses of ACF nutrition programmes (Puett et al., 2013b, 2014). A peer‐reviewed methodological guideline and self‐training module were produced to raise awareness and understanding of these methods within the organisation. Based on the continued relevance of CEA for research and programming, ACF has continued to apply these methods in research projects implemented by multiple headquarters in France, the United Kingdom, and the US (see, for example, Fenn et al., 2015; Sibson et al., 2015; Tonguet‐Papucci et al., 2015).

### Methodological objectives

The overarching analytical aim of the costing methods described in this paper is to assess and understand the total resource use of an intervention, estimating all costs that could influence intervention outcomes; this helps to highlight inefficiencies, and to understand how costs are shared among stakeholders. This objective is accomplished in several ways. First, a societal perspective is adopted, considering costs to all relevant stakeholders (Russell et al., 1996; Tan‐Torres Edejer et al., 2003), including institutions (ACF and any implementing partners) and, where feasible and relevant, communities (beneficiary households and other community members or leaders involved in the intervention). Second, mixed methods are employed, combining quantitative data from accounting records and other financial documents, and qualitative data from discussions with programme staff and beneficiaries, to produce an estimate of programme costs that reflect actual implementation and beneficiary experience participating in the intervention. Finally, an activity‐based costing framework is applied to structure and analyse costs. This enables accurate allocation of costs to programme activities, including shared support costs, and an assessment of resource use that is linked to programme implementation.

### Data collection

#### Cost data

Data on institutional costs are collected from multiple sources. First, accounting databases are reviewed with finance staff to identify all costs relevant to implementation and support of an intervention, which may be allocated across different budgets. Support costs include resources such as field office running costs, and from departments such as administration and logistics. These are an important consideration in cost analysis of humanitarian programmes. While donors consider these to be ‘indirect’ costs, they are also essential for programme functioning, particularly in complex humanitarian settings. The cost analysis therefore accounts for the opportunity cost of these support resources, since they could have been used for other purposes in the absence of the intervention under analysis.

Some relevant costs may not be included in programme accounting records, including the cost to store food supplements in the capital office or donated community space; these ‘off‐budget’ costs are identified during key informant interviews and estimated using organisation expense records or interviews. Any costs not located in accounting records are estimated using an ingredients approach (Tan‐Torres Edejer et al., 2003).

During data collection, an informal group discussion is conducted with field office staff, to understand better activities comprising the intervention, and to develop a comprehensive list and definition of each activity. After the group discussion, individual staff members are interviewed about their own experience of implementing the intervention, and about their time allocation within the programme.

Separate data collection tools are developed to capture costs borne by other implementing partners, including government ministries and community organisations, as well as costs to populations participating in the intervention. Partner costs are estimated via a review of accounting records, where possible; since partners are not always willing to share institutional accounting records, key informant interviews are held to procure estimates of unit costs and quantities of resources used during implementation to enable an ingredients approach. For beneficiary costs, tools are designed to capture direct costs, such as the costs of medicines, transportation, and food purchased to participate in an intervention, as well as the indirect costs of time spent by household members accessing care (Russell, Fryback, and Sonnenberg, 1999; Musgrove and Fox‐Rushby, 2006).

#### Effectiveness data

Researchers engaged in CEA are independent of the teams conducting individual field studies, and thus are not involved in collecting data related to programme outcomes. This helps to avoid potential conflicts of interest. Hence, CEA researchers depend on outcome data supplied by study teams assessing programme outcomes.

### Data analysis

#### Cost analysis

Institutional costs undergo several adjustments before estimates are finalised. For capital items, or large equipment such as computers and vehicles, costs are adjusted to reflect the portion of the item's value used during the intervention, typically reflected in the years it was used by the programme. This is done via amortisation, a process that allocates the cost of the item over a period of time. Costs in multi‐year interventions are adjusted for inflation, to ensure that those incurred in different years are presented in prices from a base year, usually the year of evaluation or the last year in which costs were incurred by the programme. Costs are included that are hypothesised to affect programme outcomes in some way, either directly via implementation or indirectly via supporting implementation. The costs of research and evaluations are excluded, as these exercises are not carried out to influence programme outcomes, but rather to observe or to document them. The costs of monitoring and evaluations conducted to improve implementation are retained.

Time allocation estimates enable an activity‐based cost analysis, which serves several functions. First, it allows the researcher to allocate costs to the intervention itself, and to different arms of the intervention, an important step in determining the incremental cost of one intervention as compared to an alternative. Second, it permits the exclusion of any staff costs dedicated to research or other activities that would not influence programme outcomes, as well as an assessment of staff time dedicated to different programme activities. The guiding logic of an activity‐based cost analysis is that people implement programmes: if more staff time is dedicated to an activity, then this activity also usually requires more support. This feature of activity‐based costing was deemed to be important for comparing similar interventions in terms of time spent on activities such as community mobilisation, which might influence programme effectiveness. This is tied less to specific humanitarian objectives, and more to a broader need for understanding and learning from implementation. Lastly, qualitative findings from discussions with staff and beneficiaries are used to contextualise quantitative cost data.

#### Cost‐efficiency and cost‐effectiveness analysis

Once cost estimates are finalised, cost‐efficiency analysis is conducted by dividing total programme costs by output measures, such as the number of programme beneficiaries, or the quantity of cash delivered. These estimates provide information on an intervention's coverage, or efficiency in delivering outputs, but not on its effectiveness in achieving successful outcomes.

Using effectiveness data, a base‐case cost‐effectiveness ratio is calculated by dividing total programme costs by successful outcomes achieved by the intervention. Average cost‐effectiveness ratios (ACER) provide an average cost per outcome, while incremental cost‐effectiveness ratios (ICER) estimate the additional cost per additional successful outcome achieved in one intervention as compared to an alternative. ICERs are useful when comparing two or more interventions, or when analysing cases (that is, of a disease) prevented in one intervention relative to another. If multiple successful outcomes were achieved, multiple CERs are calculated to give a range of cost‐effectiveness estimates across different outcomes. This can yield insight into whether an intervention might affect some indicators more than others, with implications for variations in cost‐effectiveness.

During sensitivity analysis, estimates of uncertainty are established for different input parameters, to gauge whether analytical outcomes (that is, CERs) change significantly given plausible levels of variation in model parameter values. For univariate sensitivity analysis, individual parameters are varied one at a time between their bestand worst‐case estimates, to determine whether variation in a specific variable strongly influences analytical outcomes. For multivariate or probabilistic sensitivity analysis, all parameters are varied together across their range of plausible values, to assess the probability that the intervention will achieve a particular level of cost‐effectiveness or ‘willingness to pay’ given uncertainty in all input parameters.

## Discussion

This paper documents methods developed to assess cost‐effectiveness of interventions implemented by a humanitarian organisation. This section discusses the insights gained, as well as the challenges encountered, in applying these methods to humanitarian interventions.

### Insights gained from applying CEA methods

An activity‐based costing approach required more time than would an analysis that employs only input‐based cost categories. This extra investment produced insights into programme implementation and component activities and into how these differed from what had been planned. Staff discussions on activity time allocation, and reflecting on how this differed from their job descriptions, was particularly valuable in the context of operational research, and considering how to resource properly research versus operations staff at different stages of the programme cycle. Group discussions with personnel revealed misconceptions and fears about CEA, with many staff members believing that the exercise was an audit of their work performance. To help allay these concerns, researchers included a sensitisation session during their field visits, to clarify the purpose of a CEA; this facilitates the process of data collection, and ensures greater accuracy of participant responses.

One challenge in applying this approach was that for some programmes with a standard set of activities, such as community‐based management of acute malnutrition or cash‐based transfers, the list of activities generated for programmes was very similar, raising questions about the relevance of the method for such programmes. Possible changes to this method in the future could include the use of standardised activity lists, while still investigating differential activity time allocation to gain potential insights into how this might affect programme outcomes.

The community‐focused approach of these methods brought insights, previously undocumented, into the cost of programme participation to households and communities. This aids programme planners in understanding better whether and how programme participation was impacted by the time burdens on households. A community garden project in Zimbabwe was time intensive for households, although this time burden was offset by income from the sale of vegetables (Puett et al., 2014). In Chad, a distribution was held during the harvest season, creating a competing interest for beneficiaries between programme participation and harvesting their own crops (Puett et al., 2013b). In addition, this analysis uncovered that time and resource burdens placed on community leaders in programme planning and preparation, such as developing and validating beneficiary lists, were onerous and created tensions locally. More broadly, the assessment of multiple cash transfer programmes revealed that high costs to households, owing, for instance, to long wait times or the cost of transportation incurred to receive the transfer, effectively decreased the value of the transfer received by poor households. If beneficiary households must decide between programme participation and their own livelihoods, or if households and communities must invest time and resources that detract significantly from their benefit received, this has negative implications for both participating communities and the quality of the programme itself; these lessons can be incorporated in the planning of future programmes.

### Better understanding of total programme costs to all stakeholders

As these methods are applied within an implementing organisation, their objective has been to understand better the total resources needed for effective interventions. These methods concentrate, therefore, on assessing the total costs to deliver an intervention, including both direct implementation and support.

There is a common apprehension that the increasing focus of donors on cost‐effectiveness could lead to a ‘race to the bottom’, favouring easily quantified, low‐cost interventions (Emmi et al., 2011). Yet, the application of ‘value for money’ to humanitarian practice is relatively new (Emmi et al., 2011), leaving room for humanitarian actors to influence its definition, especially how ‘value’ is defined, and which costs are included. Given the focus on understanding better the full scope of resources needed to enable effective intervention, the methods described in this paper do not aim to create cost estimates that make a programme appear inexpensive, that is, for advocacy purposes. Instead, in this context, resource investment is judged in terms of whether it is adequate in quality and quantity to achieve successful outcomes.

Different analyses have different aims, and other adaptations of costing methods do not account for full resource use and may concentrate instead on a subset of costs, such as those allocated to one particular donor or institutional stakeholder. It is a well‐documented concern that different methods produce cost estimates that are not comparable (Fiedler and Puett, 2015). The practical danger in costing studies is that ‘quick and dirty’ assessments probably produce more attractive ‘cheaper’ outcomes, as compared to more detailed studies. This conundrum may create a disincentive for researchers to take the time to understand full intervention resource use. Furthermore, having widely varying cost estimates can create confusion among donors and policymakers as to which one accurately represents resource requirements. It is easy to imagine that the lower cost estimate would gain more traction, thus perpetuating the problem of under‐resourcing interventions.

To some extent, these dangers can be attenuated if donors and policymakers are sensitised to these important implications of different study designs. Moreover, researchers conducting cost analyses should report transparently which costs are included in and excluded from their analyses, as well as any adjustments made to their cost data (Bulti et al., 2015). However, there is also a need to understand better the cost structures and resources required for the delivery of humanitarian interventions (Scott, 2015); this is facilitated by a thorough documentation of costs.

### Developing relevant and feasible costing methods for humanitarian programming

The CEA methods outlined here were adopted from previous research (Puett et al., 2013a) employing the ingredients approach, with cost estimates constructed from unit costs and quantities. While the ingredients approach enables transparent cost estimates (Tan‐Torres Edejer et al., 2003), it may result in an underestimation of programme costs, since it is not always possible to anticipate the full range of resources involved in programme implementation, particularly in volatile humanitarian settings. To address these important disadvantages, and to make use of routinely available cost data, the methods were adapted to utilise institutional accounting data where possible and to adjust this where necessary (such as for amortisation and inflation) to reflect accurately total programme resource use for economic analysis. Accounting information is further supplemented by key informant interviews with staff, partners, and community members to ensure it is complete.

There are some instances when it is not possible to use routinely available data, especially in societal cost analyses. Additional data collection efforts usually are required for gathering beneficiary and partner costs, and these are estimated separately using an ingredients approach. Although it may not be feasible or relevant to procure this additional data in all cases, costs borne by households participating in an intervention (and in some cases financial benefits generated by an intervention) have been found to be important boosters (Puett et al., 2014) and barriers (Puett et al., 2013a) to participation, and can provide important information to advocate for changes in policies and programmes.

### Diverse outcome measures in CEA of humanitarian programming

To assess cost‐effectiveness, it is necessary to have a measure of programme effectiveness vis‐à‐vis specific measured outcomes. The question of which effect measure to use is an important one in any CEA, and particularly in humanitarian action where outcomes can range from, for example, quantifiable anthropometric measures of nutrition status or disease outcomes, to improvements in food consumption and dietary diversity as measured by food security‐related scores, to qualitative measures of community cohesion, to name but a few.

For interventions addressing specific health outcomes, the choice of outcome measure for a CEA is relatively straightforward. Where possible, these measures can be used to calculate composite measures such as DALYs, and to compare across programmes the cost of reducing the burden of disease. However, many humanitarian programmes have multiple objectives and therefore multiple potential outcomes, and to focus on health outcomes would produce an incomplete measure of programme effectiveness. For instance, an analysis of a programme in Zimbabwe, which used community vegetable gardens to improve the nutrition and food security outcomes of people living with the human immunodeficiency virus (HIV), employed tangible measures of household dietary diversity and food consumption (Puett et al., 2014). Other intangible benefits, such as relief from social isolation and reduced stigma, could not be quantified directly as part of the CEA.

This issue of incomplete outcome measures is a common challenge in CEAs. Selecting one outcome and attributing to it all costs of an intervention will influence whether the intervention is deemed to be cost‐effective, and can lead to conclusions that are inaccurate or at least limited in scope. Currently, though, there is no comprehensive measure for non‐health outcomes, and existing measures—such as to determine particular dimensions of quality of life to enable estimation of QALYs—may still be too limited to capture the full spectrum of benefits generated by many humanitarian programmes. To help ameliorate to some extent the potential issues arising owing to incomplete outcome measures, in the methods outlined in this paper, qualitative data are used from discussions with communities and staff to provide a counterpoint to the quantitative information presented by outcome data. This practice gives weight to the perspectives of beneficiary communities as well as implementers who have seen directly myriad benefits (as well as challenges) emerge from an intervention in the field. This consideration of diverse perspectives is an uncommon approach in standard economic analysis and one that lends itself to accounting for multiple definitions of an intervention's value. These are important considerations for humanitarian programmes given their underlying principles. However, given the general interest in end‐line economic results and cost‐effectiveness ratios, the dangers of reductive interpretation without attention to context do persist.

A relevant impact to consider in humanitarian action is the lives saved by an intervention. One advantage of CEA relative to other alternative methods of economic analysis, such as benefit–cost analysis (BCA), is that it avoids placing a monetary value on human life. Findings are expressed as the cost of the intervention per life saved rather than valuing programme costs relative to the monetary value of programme benefits or outcomes, as would be done in a BCA. Aside from the methodological complexities of assigning monetary value to human life (Alderman, Behrman, and Puett, 2017), in humanitarian action the ethical challenges of such an approach are particularly salient.

Discussion of the performance and achievements of an implemented intervention ignores the important point that there is an opportunity cost to not intervening, such as potential lives lost, or lives affected by disease, famine, or war, which could have been avoided by intervening. This represents an important economic argument in favour of humanitarian intervention generally.

Finally, while the first two CEAs applying these methods were conducted retrospectively on previously implemented interventions (Puett et al., 2013b, 2014), subsequent analyses have focused on collecting cost data alongside ongoing studies, entailing its own set of risks. Usually a CEA would be conducted to answer the question ‘if this programme is effective, is it also cost‐effective?’. Consequently, a CEA requires a successful outcome relative to the comparator on one or more outcomes of interest. It is not possible to predict from the outset, though, whether an intervention will be effective relative to the comparator. For studies conducted within ACF, if there is no effectiveness finding to use for a CEA, a cost‐efficiency analysis typically is conducted instead, alongside the activity‐based cost analysis, both to document total programme costs and to provide cost per programme output instead of outcome or impact. Given the current limited evidence on the costs of humanitarian programming in general, even if no cost‐effectiveness evidence is generated, these analyses are thought to make an important contribution to the evidence base.

### Applications in operational research settings versus routine field operations

These methods are presently applied in operational research studies; concentrating on these settings brings benefits and drawbacks. Research settings have the benefit of producing reliable measures of programme effectiveness. Furthermore, research projects tend to focus on interventions of strategic interest, such as whether changing key aspects of programme delivery leads to improvements in effectiveness. Adding a CEA to such studies generates useful information on the resource requirements of such changes. For example, recent analyses have assessed how the cost‐effectiveness of community‐based management of acute malnutrition might be improved with the delivery of protocols by community health workers rather than by humanitarian staff, or by adding a water treatment component to help address health‐related underlying causes of acute malnutrition. However, in research settings, the number of beneficiaries often is lower than what would be anticipated at scale, which can limit researchers' ability to extrapolate the results of such analyses to scaled‐up programmes, an important consideration for understanding sustainability and cost‐effectiveness. In addition, given the planned nature of research, it is more feasible to assess emergencies that are predictable and cyclical in nature, such as seasonal hunger gaps (Fenn et al., 2015; Sibson et al., 2015; Tonguet‐Papucci et al., 2015), rather than less predictable or less stable conflict environments. While research in such settings entails its own challenges (Goyet et al., 2006; Ford et al., 2009), there is also less cost‐effectiveness evidence from these contexts. In applying these methods, efforts are made to exclude inputs and staff time dedicated specifically to research activities (Puett et al., 2013b). Revisions to these methods may be considered in the future if CEAs will be conducted in more routine programme settings, or emergencies, outside of a research context. Other considerations would also apply for accurately measuring effectiveness in such locations where access may be limited and quality of data poor or difficult to ascertain.

### Limitations of the methodology

The limitations of these methods are similar to those for any CEA in that context‐specific differences among interventions and study settings restrict comparability of results (Hallam, 1996; Fiedler and Puett, 2015). The strength of this approach is that in using these methods consistently, there is an opportunity to standardise the process of data collection and analysis, promoting comparability as much as possible. Furthermore, having good access to field staff to crosscheck assumptions and ensure that they are grounded in field realities is an important strength of conducting these analyses within a humanitarian organisation, given the number of often critical assumptions made during any economic analysis.

These methods were developed for application within an implementing organisation, to understand better the resource use of specific humanitarian interventions. Other kinds of humanitarian organisations, including donors, are also increasingly interested in assessing cost‐effectiveness, particularly in terms of benchmarking and comparing cost per output or outcome across different programmes and different implementing organisations (Puett and Salpéteur, 2018). Costing and CEA can serve a positive purpose in building the evidence base for economic value and resource requirements for humanitarian interventions. However, a comparison of unit costs across different interventions, disregarding context and methodological differences, represents a potentially troublesome application of the method, particularly if these comparisons are used to assist in reductive and decontextualised decision‐making on resource allocation. Caution is warranted in adopting this approach at a large scale across the humanitarian sector.

The approach outlined in this paper is novel in that it incorporates specific methodological elements that can help to expand the definition and utility of cost‐effectiveness and value for money for humanitarian action. These methodological elements include potential areas for innovation, related to new definitions of value beyond a donor‐driven agenda, by conducting analyses from the beneficiary as well as the institutional perspective. In the described methods, value is also defined via a combination of quantitative and qualitative methods, including perceptions of implementing agents, helping to counteract overly simplistic judgements of programmes based on bottomline costs alone. The regular application of such methods provides an opportunity to apply this data more broadly for assessment and improvement of humanitarian action.

## Conclusions and future directions

While there is a paucity of evidence on the cost‐effectiveness of humanitarian action, there is also increasing interest in improving accountability and transparency of mounting aid expenditure. For the past few years, ACF, a humanitarian organisation, has been engaged in developing methods to assess the cost‐effectiveness of its field programmes. Cost‐effectiveness is only one element of programme performance, and is considered a secondary analysis for programmes of strategic interest, to build the evidence base for humanitarian interventions. This information will need to be balanced with other factors, such as effectiveness and equity, which are central to humanitarian decision‐making.

These methods were developed in response to limited use and a lack of understanding of this topic within the humanitarian community, and limited evidence on the cost‐effectiveness of humanitarian action. It is hoped that sharing these methods will advance understanding of cost‐effectiveness analysis, stimulate discussion around them, and promote the adoption of cost‐effectiveness methods more broadly in humanitarian action, including assessment of beneficiary costs, to enable analytical outputs that are useful for managers and policymakers alike.

## Acknowledgements

The author would like to thank the following individuals at ACF for their interest and support in institutionalising these methods: Myriam Aït‐Aïssa, José Luis Álvarez Morán, Claire Baudon, Saul Guerrero, Anne‐Dominique Israël, Karen Martínez, Silke Pietzsch, Cécile Salpéteur, and Lani Trenouth.
